# Necrotizing Fasciitis: Diagnostic Challenges in a Mute Bedridden Patient with Atypical Laboratory Parameters

**DOI:** 10.1155/2012/253906

**Published:** 2012-11-04

**Authors:** Ghan-Shyam Lohiya, Lilia Tan-Figueroa, Vijay Krishna, Sunita Lohiya

**Affiliations:** ^1^Department of Public Health, Fairview Developmental Center, Costa Mesa, CA 92626, USA; ^2^Fairview Developmental Center, Costa Mesa, CA 92626, USA; ^3^School of Medicine, St. George's University, Grenada, West Indies; ^4^Royal Medical Group, 1120 W. Warner Avenue, Santa Ana, CA 92707, USA

## Abstract

A 27-year-old mute bedridden patient required parenteral corticosteroids and antibiotics, and hospitalization for an acute respiratory illness. After 2 days, staff noted a ~0.3 cm blister on the patient's right heel. Within 19 hours, blistering increased and the foot became partly gangrenous. The patient developed high fever (40.3°C), and leukocytosis (count: 13 × 10^9^/L; was 6.5 × 10^9^/L ten days earlier). Necrotizing fasciitis (NF) was diagnosed and treated with emergency leg amputation. Histopathology revealed necrosis of fascia, muscle, subcutaneous tissue, and skin. In bedridden patients, corticosteroids may particularly facilitate serious infections, and initial NF blistering may be mistaken for pressure ulcers. Vigilant and frequent whole body monitoring is necessary for all patients incapable of verbalizing their symptoms.

## 1. Introduction

Necrotizing fasciitis (NF) is an unusual but life-threatening bacterial infection which produces widespread necrosis of fascia, subcutaneous tissue, and skin. Its hallmark is intense local pain and tenderness, and rapid progression [1–7]. Awareness of its initial manifestations, high index of suspicion, and prompt surgical debridement are essential for better outcomes. Early diagnosis can, however, be difficult in mute bedridden patients. We illustrate this clinical challenge in a patient who required leg amputation for NF. 

## 2. Case Presentation

Our patient lived in a long-term-care facility for ~400 residents with severe developmental disabilities. He was 27 years old and had profound mental retardation, spastic quadriplegia, and permanent tracheostomy and gastrostomy. His height was 147 cm (4′10′′), weight 48 Kg (106 lbs), and body mass index 22 (normal 18–25) Kg/SqM. He had 2 NF risk factors: frequent corticosteroid treatment for respiratory illnesses during 8 of the previous ten years, and bedridden status. He did not have diabetes mellitus, significant antecedent surgery or trauma, immunodeficiency, venous ulcer, obesity, cancer, senility, or history of smoking or alcoholism. 

On day 1 of this illness, the patient developed pulmonary congestion, cough, viscid white-yellow sputum, and fever (38.2°C). Chest radiograph revealed no pneumonia. He was treated with 40 mg methylprednisolone IM for presumed viral bronchitis. 

On day 2, the patient developed greater fever (39.4°C), respiratory distress, and greenish sputum. His leukocyte count increased to 8.5 × 10^9^/L (was 6.5 × 10^9^/L 10 days earlier). Bacterial superinfection was suspected and antibiotic therapy was planned. However, no suitable vein could be found due to severe joint contractures and venous flimsiness. An IV line was eventually started in his left foot, but it soon extravasated. As temporization, treatment was begun with 250 mg levofloxacin via gastrostomy at ~10 am. 

Around 3 pm, the patient required hospitalization because of severe respiratory distress. He was given 40 mg methylprednisolone IM and 500 mg levofloxacin via gastrostomy. A central venous line was finally inserted in his left subclavian vein. He was then treated with IV vancomycin and ceftriaxone. Bronchoscopy revealed only mucosal inflammation. 

**Figure 1:**
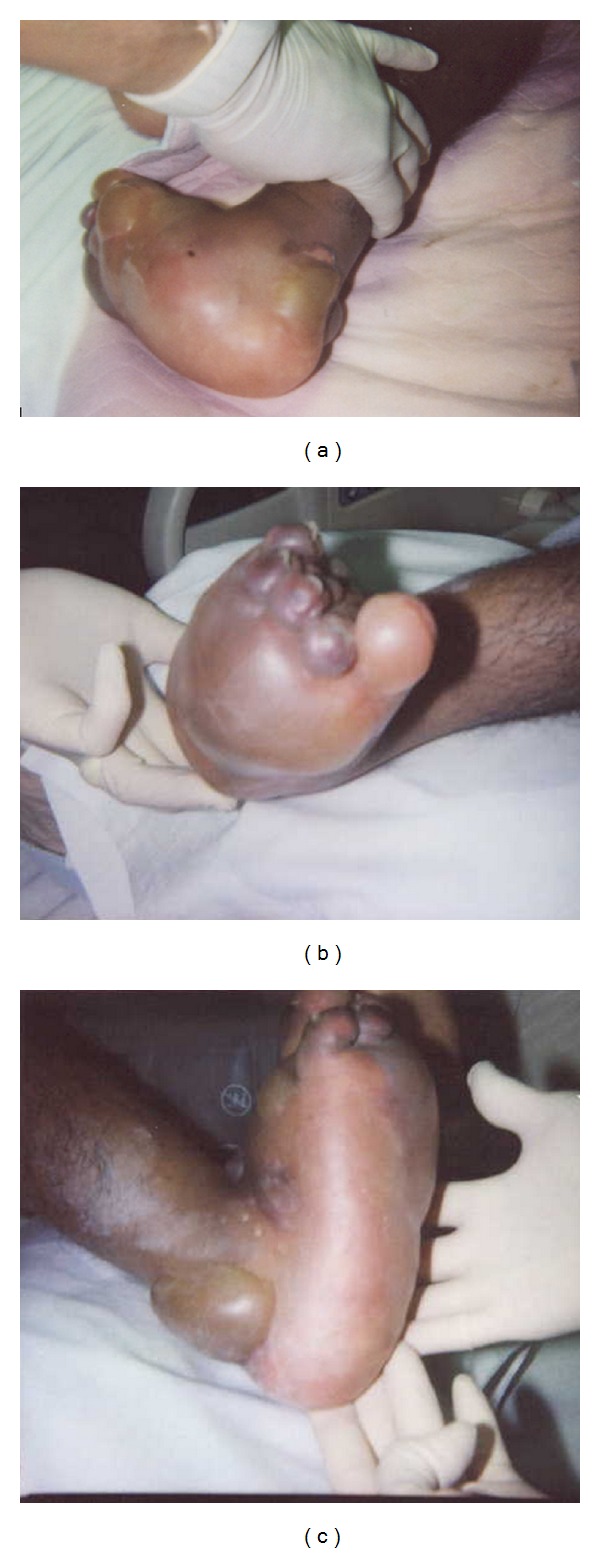
Multiple views of the patient's right foot demonstrate severe blistering and gangrene.

On day 3, the patient's pulmonary condition improved sufficiently to consider discharge planning. However, at 4 pm, during a routine whole body examination an astute employee noted a tiny (~0.3 cm) blister on the patient's posterior right heel. A stage-2 pressure ulcer was suspected and managed accordingly. Within 4 hours, however, this blister's diameter enlarged to ~3 cms. During the ensuing 12 hours, additional large blisters were formed on the foot's dorsum, sole, and the toes (Figure 1).

On day 4 around 8 am, the patient had higher fever (40.3°C) and leukocytosis (count: 13 × 10^9^/L). His right foot and toes became partly bluish black (gangrenous), with subcutaneous emphysema, edema, and increased warmth. Nevertheless, he exhibited no discomfort and remained blasé when his foot was palpated to elicit tenderness. Foot X-ray or CT scan was not performed. NF was diagnosed clinically. To prevent further NF progression, an emergency below-the-knee leg amputation was performed at 11 am (19 hours after the initial blistering). Intraoperative findings revealed massive tissue edema, blistering, desquamation, and copious serosanguineous fluid in the subcutaneous and subepithelial tissues. Microscopically, the amputated leg exhibited necrosis of the epidermis, dermis, fascia, and underlying skeletal muscle (patchy): epidermal separation from dermis, polymorphonuclear infiltrate in necrotic skin, mixed inflammatory cell infiltrate in viable tissues, acute vasculitis, and vascular thrombosis. The anterior and posterior tibial arteries were patent.

Tracheal aspirate from day 1 grew proteus mirabilis, providencia stuartii, and beta-hemolytic nongroup-A streptococcus. Bronchial washings from day 2 grew group-G beta hemolytic streptococcus. Right leg swab from day 3 grew enterococci and providencia stuartii. Blood cultures from days 2, 4, and 6 were sterile. 

The patient recovered and was discharged after a 20-day hospitalization.

## 3. Discussion

While receiving treatment for an acute respiratory illness, our patient suddenly developed a blister on his right foot. Rapidly, despite antibiotic treatment, his foot became severely blistered and partly gangrenous. NF was diagnosed clinically and required emergency leg amputation 19 hours after initial blistering. 

No wound to allow bacterial entry was identified in the patient's right foot, but there are three possibilities to explain the causation of NF. First, NF may develop following trivial wounds sustained during grooming or transfers [1]. Patient habitually rubbed his heels against bed sheets while supine, and such friction could have chafed his skin. A scar on his posterior right heel was reminiscent of a prior rubbing-related wound (Figure 1(a)). Second, although the affected foot had no IV line, venipuncture might have been attempted in it. Finally, the patient could have developed idiopathic NF without a local wound, as happens in 50% of cases [1–7].

To objectively determine the likelihood of NF among cellulitis cases, the Laboratory Risk Indicator for Necrotizing Fasciitis (LRINEC) score is commonly used (Scoring; 6-7: suspicious NF, >7: probable NF, maximum score: 13) [8]. In this patient, the specific LRINEC component values and scores were as follows: total white cell count 13 × 10^9^/L (score: 0; normal range: 4–11 × 10^9^/L), hemoglobin 117 g/L (score: 1, normal range: 14–18 g/L), sodium 140 mMol/L (score: 0; normal range: 135–145 mMol/L), creatinine 0.6 mg/dl (score: 0; normal range: 0.6–1.2 mg/dL), glucose 95 mg/dl (score: 0; normal range: 60–110 mg/dL), and C-reactive protein (not determined). For the 5 available LRINEC test results, the patient's LRINEC score was only 1. The maximum possible score for a very high C-reactive protein level (>150 mg/L; normal range: 0–5 mg/dL) is 4. Clearly this patient's LRINEC score could not have reached the NF threshold (score > 6) even if his C-reactive protein level had been determined and found very high. Thus, LRINEC was not diagnostically useful in this atypical case with only mild hematological changes and no significant metabolic impact. This observation reaffirms the wisdom of an age-old medical dictum that instructs us to rely more on clinical findings than on laboratory values! 

Bacteriologically too, this case was atypical. The usual NF initiators (group-A beta hemolytic *Streptococcus* and *Staphylococcus aureus*) were absent. Although Streptococci grew, they were not of Group-A subtype. There was growth of only facultative, but not strict, anaerobes. 

Corticosteroids are miraculously potent medicines, but they also predispose patients to infections because of their anti-inflammatory actions [9]. Bedridden patients with tracheostomy or gastrostomy are inherently infection prone because of impaired protective reflexes and underlying conditions. Corticosteroids, sometimes indispensable and life-saving, are therefore a particularly dangerous double-edged sword in this population and should be prescribed only after a thorough risk-benefit analysis.

Several factors delayed NF diagnosis in this patient. First, due to patient's mutism the cardinal NF manifestations (intense pain, severe tenderness) could not be recognized. Although usually deemed an objective sign, tenderness is partly subjective since it requires the patient's response to pressure; therefore, it may not be a useful sign in debilitated mute individuals. Second, the patient's severe deformities and contractures interfered with recognition of inflammatory edema. Third, due to patient's bedridden state and occurrence of blistering at a pressure point, his condition was initially confused with pressure ulcer [10]. Fourth, NF's rarity caused it to further elude diagnosis while blistering progressed during 16 hours of the evening and night shifts. 

Onset of blistering in cellulitis cases should automatically arouse a suspicion of NF, and the need for emergency care in the intensive care unit. Blisters form because of bacterial synthesis of gases of poor water solubility (hydrogen, nitrogen, hydrogen sulfide, and methane) [11]. Early diagnosis and prompt surgical debridement following the initial blistering might have saved patient's leg [1].

NF is usually associated with Systemic Inflammatory Response Syndrome (asthenia, palpitation, body ache, nausea, fatigue, fever, and malaise) [1]. These discomforting symptoms are actually blessings in disguise as they instigate patients to seek medical care and thus facilitate an early diagnosis. However, such “blessing” may be of no value to mute and debilitated patients like ours who can not verbalize their symptoms. Many previously able-bodied patients may also likewise become incapable of “partnering” with their physicians due to mental obtundation from serious illness or medicines. 

A diligent employee probably saved our patient's life by inspecting his right foot. Otherwise, NF diagnosis could have been further delayed as there was no clinical reason to monitor the patient's right foot. The patient was hospitalized for a nonfoot problem, and even the transient IV line had been placed in the other foot. Gait anomaly could not have provided a clue since the patient was bedridden. This case thus highlights the broader need for vigilance in the care of people with severe disabilities. The fact that NF-type complications are rare in our residents is a tribute to our staff's dedication and professionalism, as highlighted earlier too [11].
